# Ancient human sialic acid variant restricts an emerging zoonotic malaria parasite

**DOI:** 10.1038/ncomms11187

**Published:** 2016-04-04

**Authors:** Selasi Dankwa, Caeul Lim, Amy K. Bei, Rays H. Y. Jiang, James R. Abshire, Saurabh D. Patel, Jonathan M. Goldberg, Yovany Moreno, Maya Kono, Jacquin C. Niles, Manoj T. Duraisingh

**Affiliations:** 1 Department of Immunology and Infectious Diseases, Harvard T.H. Chan School of Public Health, Boston, Massachusetts 02115, USA; 2 Department of Biological Engineering, Massachusetts Institute of Technology, Cambridge, Massachusetts 02139, USA; 3 Division of Gastroenterology, Hepatology and Nutrition, Boston Children's Hospital, Boston, Massachusetts 02115, USA

## Abstract

*Plasmodium knowlesi* is a zoonotic parasite transmitted from macaques causing malaria in humans in Southeast Asia. *Plasmodium* parasites bind to red blood cell (RBC) surface receptors, many of which are sialylated. While macaques synthesize the sialic acid variant *N*-glycolylneuraminic acid (Neu5Gc), humans cannot because of a mutation in the enzyme CMAH that converts *N*-acetylneuraminic acid (Neu5Ac) to Neu5Gc. Here we reconstitute *CMAH* in human RBCs for the reintroduction of Neu5Gc, which results in enhancement of *P. knowlesi* invasion. We show that two *P. knowlesi* invasion ligands, PkDBPβ and PkDBPγ, bind specifically to Neu5Gc-containing receptors. A human-adapted *P. knowlesi* line invades human RBCs independently of Neu5Gc, with duplication of the sialic acid-independent invasion ligand, PkDBPα and loss of PkDBPγ. Our results suggest that absence of Neu5Gc on human RBCs limits *P. knowlesi* invasion, but that parasites may evolve to invade human RBCs through the use of sialic acid-independent pathways.

## Abstract (web summary)

*Plasmodium knowlesi* infects macaques and can cause malaria in humans. Here, Dankwa *et al*. show that the absence of a sialic-acid component on the surface of macaque red blood cells (RBCs) limits infection of human RBCs with *P. knowlesi*, but the parasite can adapt to invade human RBCs by using alternative pathways.

## Graphical abstract

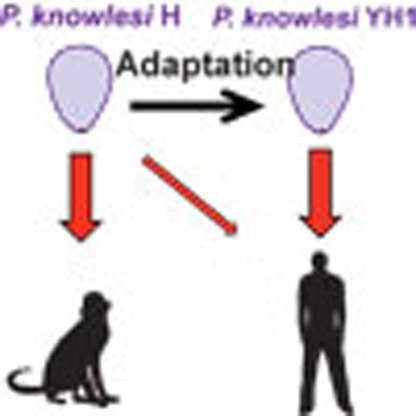

Diverse pathogens including bacteria^1^,^2^, parasites^3^,^4^ and many different viruses^5^,^6^ take advantage of the ubiquity of sialic acids on the surface of mammalian cells to attach to and infect host cells^6^. Sialic acids are a family of acidic sugars usually found at the termini of glycan chains on proteins and lipids^7^. The most common forms of sialic acid on mammalian cells are *N*-acetylneuraminic acid (Neu5Ac) and *N*-glycolylneuraminic acid (Neu5Gc). Humans express Neu5Ac, but lack Neu5Gc because of an *Alu*-mediated exon deletion in the gene encoding cytidine monophosphate Neu5Ac hydroxylase (CMAH), which converts Neu5Ac to Neu5Gc^8^,^9^,^10^. This *CMAH* gene inactivation occurred ∼3.2 Myr ago^11^, around a time of active hominin evolution^12^, and is shared by Neanderthals^13^, extinct hominins closely related to humans.

In contrast to the human lineage, CMAH is functional in great apes. Variation between human (Neu5Ac) and great ape (Neu5Gc) sialic acid was posited to underlie the host specificity exhibited by the evolutionarily related *Laverania* parasites, *P. falciparum* and *P. reichenowi*, which infect humans and chimpanzees, respectively^14^. However, recent work has challenged the view that Neu5Gc is a key host determinant of the tropism exhibited by *P. falciparum* and other *Laverania* parasites^15^,^16^.

*P. knowlesi* is unique among the human malaria-causing parasites because it displays significant cross-species transmission. *P. knowlesi* chronically infects Neu5Gc-positive long-tailed and pig-tailed macaques, as well as Neu5Ac-positive humans^17^. Most human blood-stage infections are mild and associated with low parasitaemias^18^, but increasing numbers of severe infections accompanied by high parasitaemias are being reported^19^,^20^. There is growing concern that this simian parasite is adapting to infect humans more efficiently. It has been shown that *P. knowlesi* can expand its preferred host cell niche by invading older red blood cells (RBCs)^21^, and this is an important factor influencing adaptation of *P. knowlesi* to the human population.

In this study, we sought to test the hypothesis that Neu5Gc is a key determinant of *P. knowlesi* tropism. We modified human RBCs by introducing Neu5Gc on the cell surface through expression of chimpanzee *CMAH* in *ex vivo*-cultured RBCs (cRBCs)^22^,^23^. We show that while Neu5Gc expression has no effect on *P. falciparum* invasion, it significantly increases *P. knowlesi* invasion. Further, both PkDBPβ and PkDBPγ, which belong to the Duffy binding protein (DBP) ligand family, bind to Neu5Gc-sialylated receptors, potentially facilitating invasion of Neu5Gc-expressing human cRBCs. In demonstrating that a human-adapted *P. knowlesi* lab strain efficiently invades Neu5Gc-expressing human cRBCs in a sialic acid-independent manner, we identify a potential host factor that *P. knowlesi* must overcome for efficient adaptation to humans.

## Results

### *CMAH* expression introduces Neu5Gc on human RBCs

**Figure 1:**
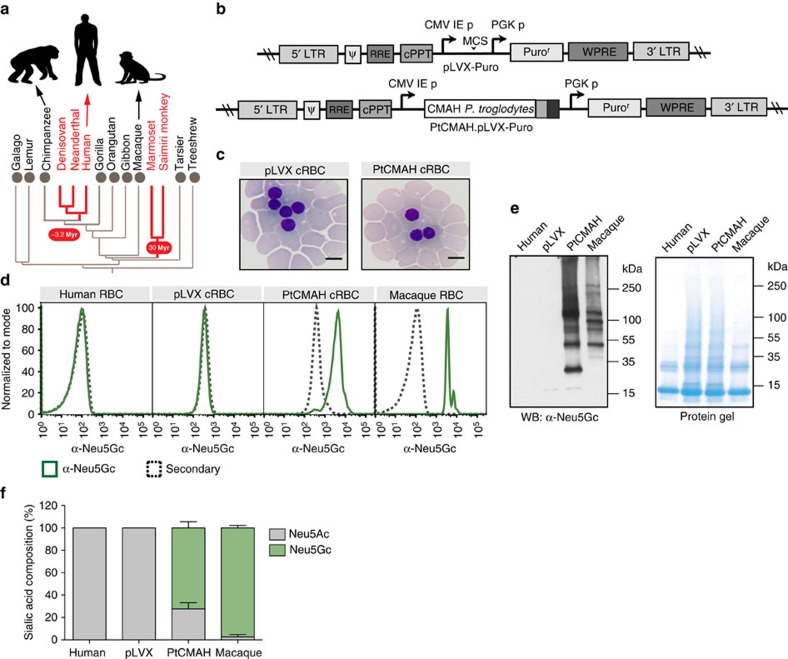
Expression of chimpanzee *CMAH* in human red blood cells (RBCs) introduces the non-human sialic acid variant Neu5Gc on the cell surface. (**a**) The phylogenetic tree for a range of primates indicates loss of *CMAH* (highlighted in red) and timing of this event in ancestral hominins and New World monkeys (marmoset and *Saimiri* monkey). Myr ago, million years ago. (**b**) Domain structures of pLVX-Puro and PtCMAH.pLVX-Puro. Ψ, packaging signal; CMV IE p, cytomegalovirus immediate early promoter; cPPT, central polypurine tract; PGK p, phosphoglycerate kinase promoter; PuroR, puromycin resistance gene; RRE, Rev-response element; WPRE, woodchuck hepatitis virus post-transcriptional regulatory element. (**c**) Normal morphology of day 21 pLVX and PtCMAH cRBCs. Scale bar, 10 μm. (**d**) Neu5Gc expression on RBCs as measured by flow cytometry using an α-Neu5Gc antibody. Shown are representative plots from at least six independent experiments. Normalized to mode—normalization to the modal fluorescence value. (**e**) Neu5Gc expression on RBCs as measured by western blot (left panel). The coomassie-stained protein gel (right panel) shows total protein from RBC samples analysed by western blot. Representative images are shown from two independent experiments. (**f**) Relative amounts of Neu5Ac and Neu5Gc on RBCs by HPLC analyses. The average of three independent experiments is shown. Error bars represent the s.e.m.

An analysis of the genomes of a large range of mammalian species indicates that CMAH loss is infrequent (Supplementary Fig. 1). The genomic sequence of a Denisovan^24^, an extinct species of the human lineage, has an exon deletion in *CMAH* like humans and Neanderthals, which would result in the loss of expression of Neu5Gc (Fig. 1a; Supplementary Fig. 2). Among primates, *CMAH* genetic inactivation has also occurred in New World monkeys^25^ (Fig. 1a, Supplementary Fig. 1), which serve as model organisms for the human *P. falciparum* and *P. vivax* parasites^26^.

To determine whether host sialic acid variation impacts *P. knowlesi* tropism, we expressed the *CMAH* gene from chimpanzee (*Pan troglodytes*) in human CD34^+^ haematopoietic stem cells by transduction, with lentivirus harbouring the chimpanzee *CMAH* transgene (PtCMAH) or an empty vector (pLVX; Fig. 1b). Terminal differentiation to mature cRBCs^22^,^23^ resulted in transgenic human cRBCs expressing PtCMAH or wild-type control cRBCs with an inactive human CMAH. Both sets of cells displayed normal RBC morphology (Fig. 1c) and had similar expression of the major glycosylated receptors, glycophorin A and glycophorin C, as well as the Duffy antigen receptor for chemokines (DARC; Supplementary Fig. 3), a critical human receptor for *P. knowlesi* invasion^27^. PtCMAH cRBCs, but not pLVX cRBCs, expressed Neu5Gc as shown by flow cytometry (Fig. 1d), and western blot analysis where we observed that Neu5Gc is added to a range of proteins of varying molecular weights (Fig. 1e). Using high-performance liquid chromatography (HPLC), we found that on PtCMAH cRBCs, Neu5Gc constitutes ∼70% of total sialic acid, while Neu5Ac is reduced to ∼30% of the amount found on pLVX cells (Fig. 1f; Supplementary Table 1), consistent with the proportion of Neu5Gc and Neu5Ac observed in chimpanzees^28^.

### Neu5Gc on human RBCs enhances *P. knowlesi* invasion

**Figure 2:**
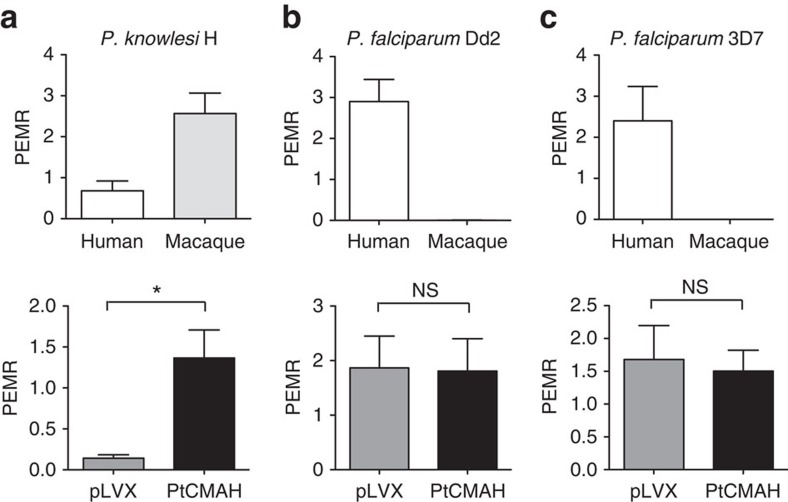
Introduction of Neu5Gc on human RBCs increases *P. knowlesi* invasion but does not affect *P. falciparum* invasion. (**a**) *P. knowlesi* H invasion of PtCMAH cRBCs is significantly enhanced, compared with pLVX cRBCs. (**b**,**c**) Sialic acid-dependent and sialic acid-independent *P. falciparum* strains Dd2 (**b**) and 3D7 (**c**), respectively, invade PtCMAH cRBCs and pLVX cRBCs similarly. Human and macaque RBCs are included as controls for each strain. The parasitized erythrocyte multiplication rate for *P. knowlesi* H is the average of four independent experiments and for *P. falciparum* Dd2 and 3D7, three independent experiments. Error bars indicate the s.e.m. The Student's *t*-test (two-tailed) was used to assess significant differences in invasion. *P=0.012. NS, not significant.

To determine the effect of human Neu5Gc expression on *P. knowlesi*, we tested invasion of the *P. knowlesi* H strain into RBCs expressing different sialic acid variants. As expected, we found that *P. knowlesi* invasion into Neu5Gc-positive rhesus macaque RBCs is more efficient than Neu5Ac-positive human RBCs (Fig. 2a). With the transgenic and isogenic cRBCs differing solely in the expressed sialic acid variant, we observed that *P. knowlesi* invades Neu5Gc-positive PtCMAH cRBCs at significantly increased levels (greater than fivefold, *P*=0.012; Student's *t*-test (two-tailed)) compared with Neu5Ac-positive pLVX cRBCs (Fig. 2a), bringing the level of *P. knowlesi* invasion close to that found for macaque RBCs. This provides direct functional evidence that the absence of Neu5Gc is a major restriction factor for *P. knowlesi* invasion of human RBCs. Our *in vitro* culture system produces cRBCs of similar age, as indicated by CD71 expression (Supplementary Fig. 3), suggesting that Neu5Gc mediates invasion independently of RBC age^21^.

### *P. falciparum* is not affected by Neu5Gc on human RBCs

To directly determine the impact of cell surface Neu5Gc on *P. falciparum* invasion, we assessed invasion of the *P. falciparum* sialic acid-dependent and sialic acid-independent laboratory-adapted strains, Dd2 and 3D7, respectively, into RBCs expressing different sialic acid variants. As expected, *P. falciparum* Dd2 and 3D7 invaded human RBCs, but not macaque RBCs^29^ (Fig. 2b,c). However, in contrast to *P. knowlesi*, both *P. falciparum* strains invaded PtCMAH cRBCs and pLVX cRBCs at similar levels (Fig. 2b,c), suggesting that *P. falciparum* can utilise both Neu5Ac and Neu5Gc. This supports the biochemical evidence that the major sialic acid-dependent invasion ligand *P. falciparum* erythrocyte-binding antigen-175 does not discriminate between Neu5Ac and Neu5Gc^15^,^16^. The PtCMAH cRBCs have ∼30% of the normal complement of Neu5Ac that might be sufficient for efficient invasion. However, this is not supported by the finding that an enzymatic reduction of Neu5Ac to ∼30% normal levels on human RBCs results in significantly decreased invasion by *P. falciparum* sialic acid-dependent lines (Supplementary Fig. 4).

### PkDBPβ and PkDBPγ bind to sialylated receptors

**Figure 3:**
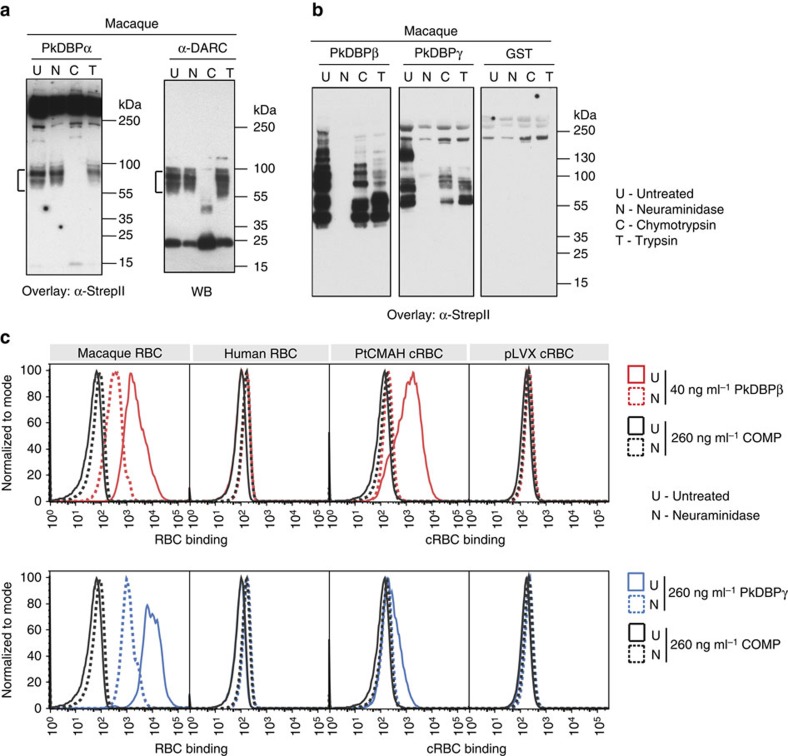
*P. knowlesi* invasion ligands PkDBPβ and PkDBPγ bind to Neu5Gc-sialylated receptors. (**a**) Left panel—PkDBPα-COMP binds to DARC on untreated (U), neuraminidase-treated (N) and trypsin-treated (T) macaque RBCs, but not chymotrypsin-treated (C) macaque RBCs as seen in a protein overlay. Right panel—α-DARC western blot confirms that the PkDBPα receptor on macaque RBCs is DARC (highlighted by the bracket). (**b**) Binding of PkDBPβ-COMP and PkDBPγ-COMP to untreated and protease-treated macaque RBCs, but not to neuraminidase-treated macaque RBCs. GST-COMP does not bind to macaque RBCs. Data are representative of at least four independent experiments. (**c**) PkDBPβ-COMP (red) and PkDBPγ-COMP (blue) bind to untreated (solid trace) PtCMAH cRBCs, as to macaque RBCs, but not to pLVX cRBCs, like human RBCs as determined by a flow cytometry-based assay. Binding to neuraminidase-treated (dashed trace) PtCMAH cRBCs and macaque RBCs is markedly decreased. COMP protein (black) does not bind to any cell type. The assay was performed twice, in triplicate; representative traces are shown.

To identify the molecular determinants of sialic acid-dependent invasion in *P. knowlesi*, we recombinantly expressed the binding domains of the *P. knowlesi* DBP ligands, PkDBPα, PkDBPβ and PkDBPγ^30^ (Supplementary Fig. 5a,b), as fusions with the cartilage oligomeric matrix protein (COMP) pentamerization domain^31^. PkDBPα binds to the DARC receptor^32^, while PkDBPβ and PkDBPγ have been shown to bind rhesus macaque RBCs^33^. We assessed binding of recombinant PkDBP domains to normal and enzyme-treated human and rhesus macaque RBC ghosts in protein overlay assays. As expected, PkDBPα did not bind to chymotrypsin-treated macaque RBC ghosts (chymotrypsin cleaves DARC). However, binding to untreated and trypsin-treated macaque ghosts, as well as macaque ghosts stripped of sialic acid by neuraminidase treatment was preserved (Fig. 3a), as expected^32^,^33^. In contrast, PkDBPβ and PkDBPγ did not bind to neuraminidase-treated macaque RBC ghosts, demonstrating that PkDBPβ and PkDBPγ are both sialic acid-binding ligands (Fig. 3b). The sialic acid dependence of PkDBPβ has been reported previously^34^, but the same study concludes that PkDBPγ binds to macaque RBCs in a sialic acid-independent manner. Our protein overlay and orthogonal flow cytometry-based-binding data (Fig. 3c; Supplementary Fig. 6) strongly suggest that PkDBPγ is also a sialic acid-dependent invasion ligand. The binding pattern for PkDBPβ and PkDBPγ to proteins within the RBC ghost overlays was different, however, implying a diversification for binding to different macaque receptors. As expected, PkDBPβ and PkDBPγ did not bind to human RBC ghosts, and glutathione s-transferase (GST) control protein did not bind to either cell type (Fig. 3b; Supplementary Fig. 5d).

### PkDBPβ and PkDBPγ bind to Neu5Gc receptors

Since PkDBPβ and PkDBPγ bound macaque but not human RBCs in a sialic acid-dependent manner, we sought to determine if binding was a function of a preference for Neu5Gc over Neu5Ac. To this end, we assayed binding of increasing concentrations of PkDBPβ and PkDBPγ to PtCMAH cRBCs, using a flow cytometry-based binding assay (Supplementary Fig. 7a). We observed dose-dependent binding of both PkDBPβ and PkDBPγ to PtCMAH cRBCs, but not control pLVX cRBCs (Fig. 3c; Supplementary Fig. 7a), suggesting that Neu5Gc is strictly required for binding. Furthermore, neuraminidase treatment of PtCMAH cRBCs abrogated binding (Fig. 3c; Supplementary Fig. 7b). Similarly, we observed Neu5Gc-dependent binding to protein receptors in cRBC overlay assays (Supplementary Fig. 7c). Together, our experiments indicate that PkDBPβ and PkDBPγ are Neu5Gc-binding ligands that can enhance invasion into Neu5Gc-expressing RBCs.

We observed stronger binding of PkDBPγ to rhesus RBCs relative to PtCMAH cRBCs potentially because of a differing linkage between the terminal sialic acid and the proximal sugar or significant differences in the structure of the protein backbone of the orthologous receptor on PtCMAH cRBCs. Both sialic acid–sugar linkage and protein backbone have been shown to be important for sialic acid-dependent ligand–receptor interactions in *P. falciparum*^35^,^36^,^37^.

### Human-adapted *P. knowlesi* invasion is Neu5Gc-independent

**Figure 4:**
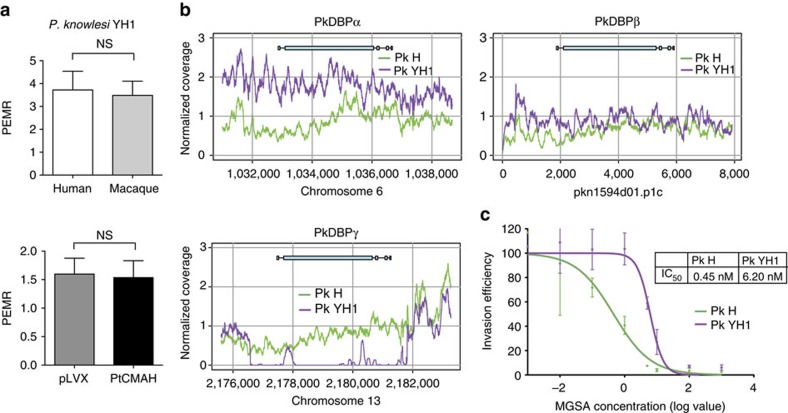
Neu5Gc-independent invasion of human-adapted *P. knowlesi* YH1 strain. (**a**) A human-adapted laboratory strain, Pk YH1, invades Neu5Gc-positive and Neu5Gc-negative RBCs similarly. Shown is the average PEMR of three biological replicates performed in triplicate. Error bars represent the s.e.m. NS, not significant (Student's *t*-test (two-tailed)). (**b**) Read coverage normalized to the average genome-wide coverage for regions containing the DBP loci in Pk YH1 and the parental Pk H strain. Pk YH1 has a PkDBPα duplication (left panel) and a PkDBPγ deletion (bottom panel). PkDBPβ in Pk YH1 is unchanged (right panel). The intron–exon structure of each gene is shown above the corresponding chromosomal region. (**c**) The IC_50_ of MGSA against Pk YH1 invasion of human RBCs is increased >10-fold compared with Pk H. Shown is the average invasion efficiency of three (Pk H) or four (Pk YH1) biological replicates. Error bars indicate the s.e.m.

Increased invasion into human RBCs could be a major factor in *P. knowlesi* pathogenesis^21^. We have adapted *P. knowlesi* strain H (Pk H) to grow efficiently in human RBCs, generating a strain named Pk YH1 hereafter^21^. To determine how Neu5Gc impacts the human-adapted *P. knowlesi* strain, we assessed invasion of Pk YH1 into PtCMAH cRBCs and pLVX cRBCs. In contrast to the parental Pk H strain, Pk YH1 invasion into PtCMAH cRBCs was not enhanced relative to pLVX cRBCs (Figs 2a and 4a). As expected, Pk YH1 invaded human and macaque RBCs equally well (Fig. 4a). These results suggest that Pk YH1 has adapted to use an altered set of functional invasion ligands for human infection, circumventing the dependence on Neu5Gc for optimal invasion.

Whole-genome sequencing of the Pk YH1 and parental Pk H strains was carried out to identify changes in invasion ligands of the DBP family and the recently described orthologues of the reticulocyte binding-like (RBL) family^38^. Sequencing revealed a duplication of the region containing the sialic acid-independent *PkDBPα* gene, as well as a complete deletion of the sialic acid-dependent *PkDBPγ* gene in Pk YH1 (Fig. 4b; Supplementary Fig. 8; Supplementary Table 2). These alterations suggest that the mechanism for the observed increase in Pk YH1 invasion into human RBCs is a shift towards greater usage of the sialic acid-independent PkDBPα ligand, and away from the PkDBPβ and PkDBPγ ligands that are unable to bind human Neu5Ac.

To test the hypothesis that Pk YH1 has increased usage of the PkDBPα sialic acid-independent ligand, we assessed the invasion of Pk YH1 and Pk H into human RBCs in the presence of increasing concentration of the chemokine melanoma growth stimulatory activity (MGSA; Fig. 4c). MGSA binds specifically to DARC and inhibits its interaction with PkDBPα^39^. We observed a greater than 10-fold increase in the half-maximal inhibitory concentration (IC_50_) for Pk YH1 (6 nM) compared with Pk H (0.5 nM) (Fig. 4c). The requirement for more MGSA for the inhibition of Pk YH1 is consistent with an increased number of PkDBPα–DARC interactions, resulting from PkDBPα amplification and loss of PkDBPγ.

## Discussion

In this study, we used an *ex vivo* RBC culture system coupled with lentiviral transgene expression of *CMAH* to specifically and functionally study Neu5Gc, as a determinant of the tropism displayed by the zoonotic macaque parasite *P. knowlesi*. We provide evidence that *P. knowlesi* is restricted in its invasion of human RBCs due to the absence of surface Neu5Gc. Evolutionarily, this modified sialic acid was once present in ancestral humans Myr ago. It is plausible that selective pressure due to a *Plasmodium* parasite utilizing this invasion pathway could have resulted in loss of a functional CMAH in ancestral humans, given the considerable evidence that malaria has shaped the human genome^4^,^40^. However, fossil records of ancestral macaques in Africa do not place them in East Africa at the site of *CMAH* inactivation, but rather in North Africa, from where they migrated to Eurasia 5.5 Myr ago^41^. Thus, an ancestral zoonotic *P. knowlesi* species is less likely to have been the selective force for CMAH loss ∼3.2 Myr ago. We speculate that a *Laverania* parasite, such as *P. reichenowi*, that infects chimpanzees (which have Neu5Gc-positive RBCs) is a more likely candidate for the selective pressure leading to CMAH loss. Regardless of the origins, we suggest that *CMAH* gene inactivation in humans is currently a restriction factor limiting the emergence of *P. knowlesi* infections in the human population.

However, the human-adapted Pk YH1 strain used in this study suggests that *P. knowlesi* can adapt to overcome this restriction through duplication of the sialic acid-independent PkDBPα invasion ligand. Our hypothesis that Pk YH1 has greater and more efficient usage of the PkDBPα–DARC invasion pathway is supported by the increased IC_50_ for Pk YH1 in the MGSA invasion inhibition assay. We also observed deletion of the Neu5Gc-dependent PkDBPγ, although whether this was a necessary change concomitant with the duplication of PkDBPα, remains to be determined.

The space-filling model for the display of invasion ligands at the apical end of the invading parasite^42^,^43^ can be one way to rationalize how increased expression of PkDBPα and loss of PkDBPγ may enhance invasion of human RBCs. With limited space at the apex of the parasite for the deployment of invasion ligands before invasion, increased expression of PkDBPα and absence of PkDBPγ would result in greater release of PkDBPα to the apex of the parasite, resulting in an increased number of productive PkDBPα–DARC interactions. PkDBPβ and PkDBPγ are expressed together with PkDBPα in wild-type *P. knowlesi*^44^,^45^, making plausible the increased apical presentation of PkDBPα in the absence of PkDBPγ in Pk YH1.

The duplication of PkDBPα in Pk YH1 is reminiscent of the duplication of the closely related *P. vivax* DBP invasion ligand observed in *P. vivax* isolates in Madagascar^46^. This *P. vivax* DBP duplication, likely a recent event, may have been selected for the efficient invasion of Malagasy individuals, where RBCs that are either homozygous for DARC-negative alleles or heterozygous for DARC-negative/DARC-positive alleles are common.

The rise in reported malaria cases due to *P. knowlesi* infection in Southeast Asia^19^,^20^ raises concern about the possible occurrence of human-to-human transmission. There is already evidence suggesting the clustering of *P. knowlesi* infections in households, where individuals had not been in proximity to macaques^47^,^48^. In the 1950s, the use of *P. knowlesi* as a treatment for tertiary neurosyphilis had to be stopped because of increased pathogenicity of the parasite, with continuous passaging in humans^49^,^50^. This suggests that increased parasite adaptation to humans may be associated with severe disease. We suggest that genetic changes in the DBP invasion ligands might be a sentinel indicator of increased adaptation to humans.

## Methods

### Investigation of *CMAH* gene loss in mammalian evolution

We examined hominid and mammalian genomes with genome alignments and gene loss analysis. The mammalian gene loss events were analysed with the program CAFE (Computational Analysis of gene Family Evolution^51^), a statistical tool for computing the gene family size evolution that is implemented in Ensembl 72. The gene loss event in hominins was further verified with independent genomic analysis; the sequence reads of the Neanderthal^52^ and Denisovan genomes^24^ were aligned to chimpanzee genome panTro3 at the *CMAH* gene locus with the University of California, Santa Cruz (UCSC) genome browser^53^.

The ferret^54^ and New World monkey^25^ *CMAH* gene loss events are published. Dog CMAH does not appear to be functional in certain breeds of dogs^55^,^56^.

### *Ex vivo* culture of RBCs

Bone-marrow-derived CD34^+^ haematopoietic stem cells (2–3 × 10^5^; Lonza) were cultured in media with 5% solvent/detergent virus-inactivated human plasma (Octaplas, Octapharma) as described previously^57^ with the following modifications. On day 6 or 7 of culture, cells were transduced with lentivirus harbouring either the chimpanzee (*Pan troglodytes*) CMAH complementary DNA (cDNA) sequence in pLVX-Puro (Clontech) or the empty vector, pLVX-Puro. The CMAH cDNA sequence with a C-terminal tobacco etch virus (TEV) cleavage site and FLAG-c-myc tags was codon-optimized for human expression and synthesized (GeneArt). CMAH cDNA was amplified using primers listed in Supplementary Table 3 and cloned into pLVX-Puro. Transduction with lentiviral vectors and subsequent selection on 2 μg ml^−1^ puromycin dihydrochloride (Sigma-Aldrich) were done as described^23^, but puromycin was maintained until day 12 or 13 when cells were co-cultured on a murine MS-5 stromal cell layer at a cell density of 3–6 × 10^5^ cells per ml until day 18 as described^58^. On day 18, cells were replated on a fresh MS-5 stromal cell layer and then collected on day 20 or 21 at which time 70–90% of cells were enucleated. Cells were stored at 4°C in incomplete RPMI; (consisting of RPMI-1640 (Sigma-Aldrich) supplemented with 25 mM 4-(2-hydroxyethyl)-1-piperazineethanesulfonic acid (HEPES) and 50 mg l^−1^ hypoxanthine), until use in downstream experiments.

### Neu5Gc or RBC receptor expression by flow cytometry

RBCs and cRBCs were washed three times in PBS containing 0.5% Neu5Gc-free blocking agent (Siamab, formerly Sialix) and pelleted at 500 *g* for 4 min in a 96-well plate at 5 × 10^5^ cells per well. Cells were resuspended in either 50 μl of blocking buffer or 50 μl of the appropriate antibody solution. The following antibodies were used at the indicated dilutions: anti-Neu5Gc (1:5,000, Siamab), phycoerythrin(PE)-conjugated anti-DARC (1:10, Miltenyi Biotec), anti-CD71-PE (1:10, Miltenyi Biotec), fluorescein isothiocyanate-conjugated anti-glycophorin A (GPA); 1:50, Clone 2B7, STEMCELL Technologies) and anti-glycophorin C (GPC)-fluorescein isothiocyanate (1:500, BRIC 10, Santa Cruz). Cells were incubated for 1 h at room temperature and washed three times in blocking buffer. Unstained cells and cells stained with DARC, CD71, GPA and GPC antibodies were resuspended in 100 μl PBS for analysis on the MACSQuant flow cytometer (Miltenyi Biotec). Neu5Gc-stained cells were incubated in anti-chicken IgY-Alexa Fluor 488 secondary antibody (Life Technologies) at 1:1,000 for 30 min at room temperature. Control samples were similarly incubated in anti-mouse IgG2a-PE (1:10, Miltenyi Biotec), anti-chicken IgY-Alexa Fluor 488 antibody or anti-mouse IgG-Alexa Fluor 488 (1:1,000, Life Technologies). Cells were washed twice and subjected to flow cytometric analysis. The data were analysed in FlowJo 4 version 10.0.7 (Tree Star).

### Measurement of sialic acid by HPLC

For measurement of cell surface sialic acid, RBC and cRBC ghosts were prepared for sialic acid release as described^59^, with some modifications. In brief, 1 × 10^7^ cells were washed once in incomplete RPMI and twice in PBS pH 7.4. Cells were stored at −80 °C until RBC ghost preparation, where thawed RBCs were resuspended in cold 5 mM sodium phosphate pH 8.0 containing protease inhibitor cocktail. RBC ghosts were pelleted at 21,130 *g* for 10 min, washed three times in PBS containing protease inhibitor cocktail and stored at −80 °C until sialic acid preparation. Sialic acid release from RBC ghosts was achieved by mild acid hydrolysis as described^60^, with some modifications. In brief, RBC ghosts were resuspended in 75 μl 2 M acetic acid and heated at 80 °C for 3 h. For formation of fluorescent derivatives, 75 μl of derivatization solution was added to sialic acid samples and heated at 50 °C in the dark for 2.5-3 h. The derivatization solution was made up of 7 mM 1,2-diamino**-4,5**-methylenedioxybenzene dihydrochloride, acetic acid (1.7 M for experimental samples or 3.7 M for sialic acid standards), 0.75 M β-mercaptoethanol and 18 mM sodium hydrosulfite. Samples were run on an HPLC with a C18 column at a flow rate of 0.9 ml min^−1^, using a mobile phase of acetonitrile–methanol–water (9:7:84 v/v). Sialic acid elution was monitored by absorbance at 373 nm. Data were acquired on Chemstation Rev A. 10.01 (Agilent Technologies). Eluted sialic acids were compared with a sialic acid reference panel, and Neu5Ac and Neu5Gc solutions of known amounts for quantitation of sialic acids.

For measurement of sialic acid in invasion assays, neuraminidase treatment of RBCs was performed in 40 μl total volume. Supernatants were collected and sialic acid was quantified as described previously^61^. In brief, cell supernatants were treated with 20 μl of a freshly prepared solution of 0.2 M sodium periodate in 48% phosphoric acid for 20 min at room temperature. To terminate the reaction, 100 μl of freshly prepared 10% sodium arsenite in 0.1 N sulphuric acid was added slowly, then vortexed until clear and incubated 5 min at room temperature. Finally, 600 μl of thiobarbituric acid (6 mg ml^−1^) was added, and the reaction was incubated at 100 °C for 15 min, then chilled on ice. Samples were analysed on the HPLC as described above, but elution was performed using a running buffer consisting of 115 mM sodium perchlorate, 30% methanol and 1% phosphoric acid, and absorbance was monitored at 549 nm.

### *Plasmodium* parasite cultures

*P. falciparum* Dd2^attB^/BSD-GFP (BSD: blasticidin S-deaminase) and 3D7^attB^/BSD-GFP^62^ used in cRBC/RBC invasion assays were kind gifts from David Fidock (Columbia University). Pk H SSUD^attB^/BSD-GFP (SSUD: small subunit of the D-type ribosomal RNA gene) was generated in our lab as follows. The targeting vector, pDEF-DSSU-hDHFR^63^, was integrated into Pk H (provided by A.W. Thomas; Biomedical Primate Research Center, Rijswijk) to form Pk H SSUD. The *P. knowlesi d-ssu* gene was then amplified using Pk H SSUD genomic DNA and primers listed in Supplementary Table 3, and then cloned into pCG6-attB^62^. The resulting vector, pSSUD-CG6-attB, was integrated into Pk H SSUD to form Pk H SSUD^attB^. In the final step, pBSD-GFP-INT-attP^62^ was integrated into Pk H SSUD^attB^. Pk YH1 was generated in our laboratory from the parental Pk H strain by growth for 3 months in human O^+^ RBCs supplemented with 16% reticulocytes purified from haemochromatosis patient blood^21^. The percentage of reticulocytes was decreased to 2–4% for an additional 2 months, after which parasites proliferated stably in the absence of reticulocyte-enriched blood. *P. falciparum* Dd2 and W2mef strains used in invasion assays with neuraminidase-treated RBCs were obtained from the Malaria Research and Reference Reagent Resource.

*P. falciparum* cultures and the human-adapted Pk YH1 line were maintained in human RBCs (Research Blood Components), while *P. knowlesi* H SSUD^attB^/BSD-GFP was grown in rhesus macaque RBCs (New England Primate Research Center). Parasites were grown at 2% haematocrit in complete RPMI (RPMI supplemented with 25 mM HEPES, 50 mg l^−1^ hypoxanthine, 2.57 mM sodium bicarbonate and 0.5% AlbuMAX II (Life Technologies)).

### Enzyme treatments

For RBC binding assays, enzyme treatments were performed as described^42^. In brief, RBCs or cRBCs were washed three times in incomplete RPMI and treated with 1 mg ml^−1^ trypsin (Sigma-Aldrich) and/or 1 mg ml^−1^ chymotrypsin (Worthington) and/or 66.7 mU ml^−1^ *Vibrio cholerae* neuraminidase (Sigma-Aldrich) at 37 °C with gentle mixing for 1 h. RBCs were then washed twice in incomplete RPMI and once in PBS pH 7.4.

For neuraminidase treatment for invasion assays, human RBCs were washed in wash media (RPMI supplemented with 25 mM HEPES-KOH, pH 7.4) and treated with decreasing amounts of neuraminidase from *Clostridium perfringens* (100, 50, 25, 12.5, 6.25 and 3.125 U; New England Biolabs) at 37 °C with gentle mixing for 30 min. The neuraminidase-treated RBCs were then washed extensively in complete RPMI before use. The neuraminidase-treated RBCs were subjected to a second neuraminidase treatment to remove and quantify the remaining sialic acid. This second treatment was performed at 250 U neuraminidase at 50% haematocrit for 30 min at 37 °C in PBS pH 6.0.

### Invasion assays

For invasion assays using RBCs and cRBCs, cells were washed in complete RPMI and counted on a haemocytometer. Cells (1.5 × 10^6^) were added to respective wells of a half-area 96-well plate at 0.5% haematocrit in triplicate. Synchronised schizont-stage parasites were purified by magnetic separation on a MACS cell separator (Miltenyi Biotec) and added to RBCs at ∼2% parasitaemia in a final volume of 30 μl. Cells (2.5 × 10^5^; 5 μl) were taken from each well for cytospin preparations to assess initial parasitaemia by light microscopy. Assay plates were kept in a modulator incubator chamber gassed with 1% O_2_, 5% CO_2_ and balance of N_2_, and reinvasion and growth were monitored over time by thin smear of mock wells. At trophozoite/schizont stage, final parasitaemia was estimated by light microscopy from counts of at least 1,000 cells on May-Grünwald- and Giemsa-stained cytospins. The parasitized erythrocyte multiplication rate (final parasitaemia (after single round of invasion)/initial parasitaemia) was determined for each strain in each cell type. The Student's unpaired *t*-test (two-tailed) was used to assess significant differences in invasion.

For invasion assays with neuraminidase-treated RBCs, parasite invasion into neuraminidase-treated RBCs was measured by SYBR Green I (Life Technologies) staining and flow cytometry as described previously^64^, with several modifications. Synchronised schizont-stage parasites at ∼10% parasitaemia that had been treated with 250 U *C. perfringens* neuraminidase at ring stage to reduce reinvasion, were washed, and then mixed with untreated and neuraminidase-treated target cells. Invasion assays were seeded in triplicate 200 μl samples at ∼1% parasitaemia at 0.5% haematocrit in a 96-well plate. To quantify inoculum parasitaemia, a subset of samples was fixed immediately with formaldehyde and stored at 4 °C. The remaining samples were incubated for 48 h, then fixed for 1 h. Inoculum and post-invasion samples were then stained with SYBR Green I and analysed by flow cytometry. Expansion rates were expressed as parasitized erythrocyte multiplication rate.

For MGSA invasion inhibition assays, human RBCs were plated in a 96-well plate at 4% haematocrit in 2 × complete RPMI (complete RPMI with twice the amount of AlbuMAX II and sodium bicarbonate). MGSA (PeproTech) of varying concentrations made up in incomplete RPMI were added to respective wells. Schizont-stage parasites purified by magnetic separation, as described above, were added to each well at ∼1% parasitaemia. After allowing for a single round of invasion, parasitaemia was measured the following day by either SYBR green I or Vybrant DyeCycle Violet (Life Technologies) staining and flow cytometry. The parasitaemia of MGSA-treated samples was normalized to a mock-treated control for each strain. The data were analysed in GraphPad Prism 5.

### Protein expression plasmids

Region II of PkDBPα (gene ID: PkH_062300) and Region II of PkDBPβ (gene ID: PkH_000490) were amplified from *P. knowlesi* H genomic DNA, while Region II of PkDBPγ (gene ID: PkH_134580) was amplified from a *P. knowlesi* fosmid obtained from a *P. knowlesi* fosmid library^65^, using primers listed in Supplementary Table 3. PCR products were cloned into the mammalian expression vector, pSDP32, a version of pTT^66^ with a modified multiple cloning site and with the rat COMP domain downstream of the multiple cloning site. For the GST-COMP-expressing plasmid, GST was amplified from pET-42a(+) (Supplementary Table 3) and cloned into pSDP32. For the COMP-negative control plasmid, NheI- and NotI-digested pSDP32 was modified with T4 DNA polymerase (New England Biolabs) to generate blunt ends and then ligated. Plasmid constructs were verified by sequencing.

### HEK293-6E culture and protein expression and purification

HEK293-6E cells, a kind gift from Yves Durocher (National Research Council, Canada), were maintained in serum-free Freestyle 293 expression media (Life Technologies) supplemented with 0.1% Pluronic F-68 (Life Technologies) and 25 μg ml^−1^ Geneticin (Life Technologies). Cells were grown in suspension in Erlenmeyer flasks at 110 r.p.m., at 37 °C in 8% CO_2_. Recombinant protein expression by transient transfection was achieved as described^66^ with some modifications. In brief, cells were transfected at a cell density of 1 × 10^6^ cells per ml using 1 μg of plasmid DNA per 1 × 10^6^ cells and a DNA:polyethylenimine (PEI) ratio of 1:3 (w/w). DNA and the transfection reagent, linear PEI (Polysciences, Inc.), were diluted separately in complete Freestyle 293 media. The DNA was vortexed for 10 min, and DNA and PEI were combined, vortexed for 30 s, allowed to incubate at room temperature for 12 min and finally added to the cell culture. Between 24 and 48 h post transfection, a solution of Tryptone-N1 (Organotechnie SAS) was added to the transfected cell culture at 0.5% (w/v). Cultures were processed on day 5 or 6 post transfection by pelleting and collecting the culture supernatant containing the expressed protein. Protein expression was confirmed by western blot using Strep-Tactin-HRP (IBA). Culture supernatant containing recombinant protein was used in protein overlay assays. For flow cytometry-based-binding assays, PkDBPβ, PkDBPγ and COMP protein were affinity-purified from culture supernatant on a StrepTactin sepharose column (StrepTrap HP; GE Healthcare) using an ÄKTAFPLC Fast Protein Liquid Chromatography system (GE Healthcare). Bound protein was eluted by 2.5 mM desthiobiotin (Sigma-Aldrich). Buffer exchange into PBS pH 7.4 was achieved using centrifugal filter units. Protein concentration was determined from absorbance measurements at 280 nm and the protein-specific extinction coefficient.

### Western blot analyses

HEK293-6E culture supernatant, RBCs or RBC ghosts were electrophoresed on 4–15% gradient gels and transferred to nitrocellulose membrane. Membranes were blocked in PBS–0.05% Tween (PBST)–10% milk (for probing with Strep-Tactin-HRP), tris-buffered saline–0.1% Tween–0.5% Neu5Gc-free blocking agent (for probing with anti-Neu5Gc) or PBST–5% milk (for probing with anti-DARC; Sigma-Aldrich). Membranes were probed with the respective antibodies at the indicated dilutions—anti-DARC: 1:1,000 in blocking buffer, anti-Neu5Gc: 1:10,000 in blocking buffer and Strep-Tactin-HRP: 1:2,500 in PBST–20 μg ml^−1^ avidin (PBST-A) after pre-incubation in PBST-A for 10 min. The DARC western blot was probed with anti-rabbit-HRP (GE Healthcare) at 1:10,000, while the Neu5Gc western blot was probed with anti-chicken/turkey-HRP (Life Technologies) at 1:5,000 in blocking buffer. All membranes were developed with SuperSignal West Pico chemiluminescent substrate (Thermo Scientific) and imaged by autoradiography.

### Protein overlay assay

Untreated and enzyme-treated rhesus macaque, and human RBC ghosts and cRBC ghosts were prepared by cell lysis, and multiple washes in cold 5 mM sodium phosphate pH 8.0. Protein content of macaque and human RBC ghosts was quantified by Bradford assay. RBC/cRBC ghosts were stored at −80 °C until use. Before use, cRBC ghosts were treated with DNase I (New England Biolabs). Samples of enzyme-treated or untreated macaque and human RBC ghosts or cRBC ghosts, not treated with reducing agent or heat-denatured, were electrophoresed; 10 μg of macaque or human RBC ghosts (corresponding to ∼3 × 10^7^ cells) were loaded per lane, while cRBC ghosts made from ∼1.7 × 10^6^ cells were loaded per lane. Electrophoresed protein was transferred to nitrocellulose membrane. Membranes were blocked in PBST-10% milk for 2 h at room temperature and then washed in PBST. HEK293-6E culture supernatant containing recombinant protein with Tween 20 added to a final concentration of 0.1% was added to membranes and incubated overnight at 4 °C with gentle rotation. Membranes were washed, blocked in PBST-A for 10 min, incubated in Strep-Tactin-HRP at a dilution of 1:2,500 in PBST-A for 1 h at room temperature and finally developed with Pico substrate. Bound protein was detected by autoradiography.

### Flow cytometry-based binding assay

RBCs or cRBCs were washed three times in PBS–0.5% Neu5Gc-free blocking buffer. Cells were pelleted in a 96-well plate at 3 × 10^5^ RBCs per well and resuspended in either 50 μl blocking buffer or PkDBPβ-COMP, PkDBPγ-COMP or COMP protein diluted in blocking buffer at the appropriate concentrations. Samples were incubated for 1 h at room temperature, washed three times in blocking buffer and incubated in a monoclonal anti-strepII-tag antibody (IBA) at 2 μg ml^−1^ for 1 h at room temperature. Samples were washed three times in blocking buffer then incubated in anti-mouse IgG-Alexa Fluor 488 antibody for 30 min at room temperature. Samples were washed twice, then resuspended in 100 μl PBS for analysis on the MACSQuant flow cytometer. Data were analysed by FlowJo 4 version 10.0.7.

### Whole-genome sequencing

Genomic DNA was extracted from schizont-stage Pk H and Pk YH1 parasites using the QIAamp DNA blood kit (Qiagen). Genomic DNA was used to construct Illumina sequencing libraries for both *P. knowlesi* strains, with a library insert size of 200 bp. Each library was sequenced to a depth of at least 50-fold coverage using 101-bp paired-end reads on an Illumina HiSeq 2000 sequencer. Reads were aligned to the reference genome (*P. knowlesi* H, PlasmoDB release 11) by applying the bwa mem algorithm from the Burrows–Wheeler Aligner^67^ to the left and right read files using default settings. Alignments were converted from SAM to BAM format, sorted and indexed using SAMtools^68^. Coverage was calculated using the BEDtools genomeCoverageBed function^69^.

Whole-genome sequencing resulted in 23,386,393 reads for Pk H and 21,200,464 reads for Pk YH1. 95.05% of Pk H reads and 88.77% of Pk YH1 reads mapped to the *P. knowlesi* reference genome. The average coverage depth for Pk H was 89.43% and for Pk YH1 74.59%. These results represent a genome coverage of 99.86% for Pk H and 99.87 for Pk YH1.

## Additional information

**Accession codes:** Whole-genome sequence data for *P. knowlesi* H and *P. knowlesi* YH1 have been deposited in the NCBI Sequence Read Archive (SRA) under accession codes SRX1175910 and SRX1553878, respectively.

**How to cite this article:** Dankwa, S. *et al*. Ancient human sialic acid variant restricts an emerging zoonotic malaria parasite. *Nat. Commun.* 7:11187 doi: 10.1038/ncomms11187 (2016).

## Supplementary Material

Supplementary Information Supplementary Figures 1-8 and Supplementary Tables 1-3
