# Supplementary material for: Ancient human sialic acid variant restricts an emerging zoonotic malaria parasite
Source: Nat Commun. 2016 Apr 4;7:11187. doi: 10.1038/ncomms11187 (PMC4822025; doi:10.1038/ncomms11187)
Supplement: Supplementary Information — Supplementary Figures 1-8 and Supplementary Tables 1-3 [file ncomms11187-s1.pdf]

Title:
Ancient human sialic acid variant restricts an emerging zoonotic malaria parasite

Authors:
Selasi Dankwa[1], Caeul Lim[1], Amy K. Bei[1], Rays H.Y. Jiang[1], James R. Abshire[2], Saurabh D. Patel[1,3], Jonathan M. Goldberg[1], Yovany Moreno[1], Maya Kono[1], Jacquin C. Niles[2], Manoj T. Duraisingh[1]*

*Corresponding author: Manoj T. Duraisingh, mduraisi@hsph.harvard.edu

Supplementary Information

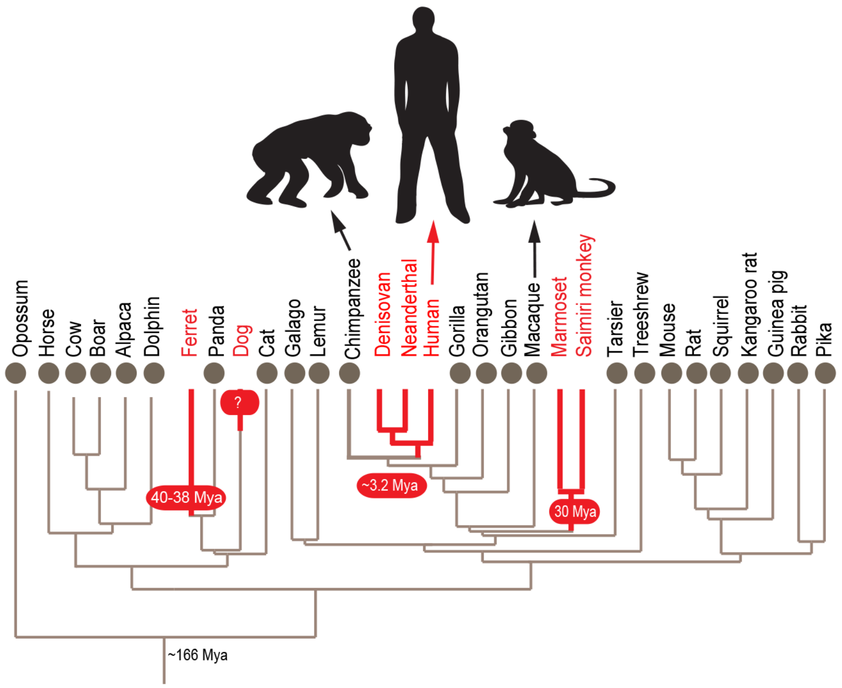

Supplementary Figure 1. Loss of *CMAH* has occurred independently in a few mammalian lineages including the human lineage. The phylogenetic tree for a range of mammalian species indicates the presence or absence of *CMAH* as determined by genome alignments and gene loss analysis (CAFE). Loss of *CMAH* is highlighted in red and dating of the loss, where known, is indicated. Mya – million years ago.

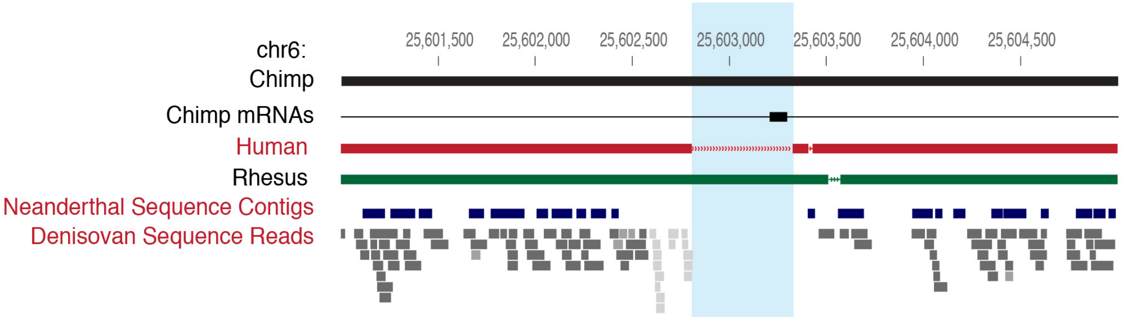

Supplementary Figure 2. Humans, Neanderthals and Denisovans share the *CMAH* exon deletion. The *CMAH* gene locus in the chimpanzee genome was used as the reference for alignments. Sequence reads of the Neanderthal genome and the Denisovan genome were aligned to chimpanzee genome PanTro3 at the *CMAH* gene locus with the UCSC genome browser. For the Neanderthal genome, sequence contigs were plotted. For Denisovan sequence reads, darker shades of grey indicate higher level of sequence similarity. All hominin sequences show a similar gap in the alignments. Chr6 – chromosome 6.

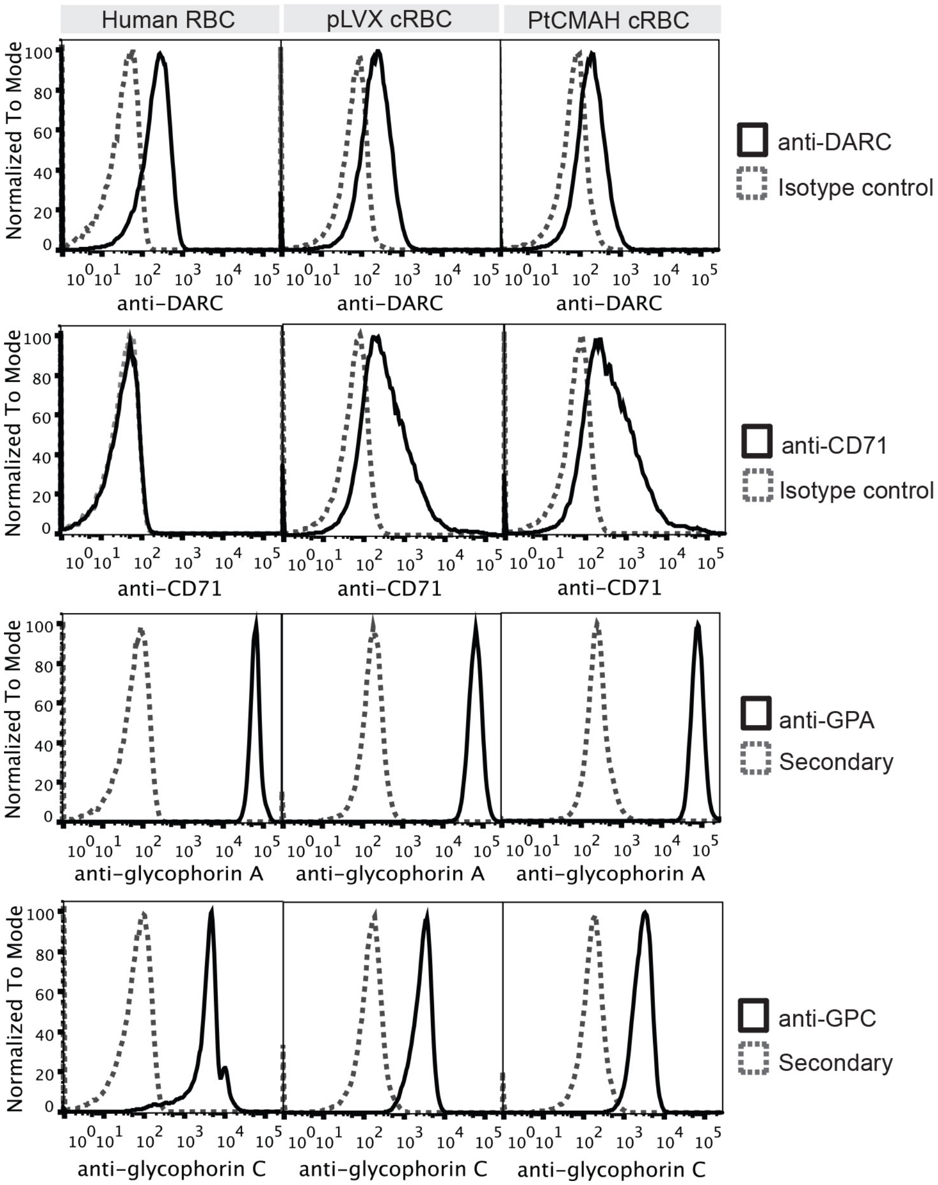

Supplementary Figure 3. Similar maturation of PtCMAH cRBCs and pLVX cRBCs. Flow cytometry plots showing expression of DARC, CD71, glycophorin A (GPA) and glycophorin C (GPC) on human RBCs and PtCMAH and pLVX cRBCs. Negative control samples were either stained with an anti-mouse IgG2a-PE antibody (isotype control) or an anti-mouse IgG-Alexa Fluor 488 antibody (secondary). Normalized to mode – normalization to the modal fluorescence value.

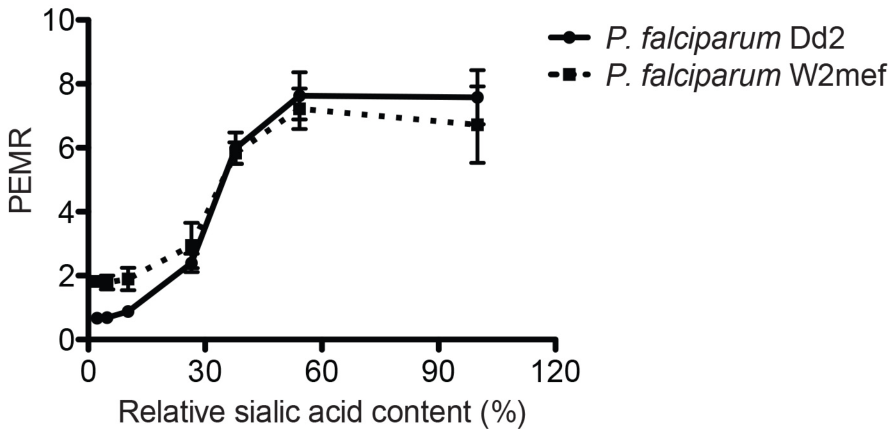

Supplementary Figure 4. Sialic acid dependence in *P. falciparum* invasion. Shown is the Parasitized Erythrocyte Multiplication Rate (PEMR) of *P. falciparum* sialic acid-dependent laboratory strains, Dd2 and W2mef, into human RBCs treated with decreasing concentrations of neuraminidase. The sialic acid remaining on RBCs after neuraminidase treatment relative to untreated RBCs is plotted. The data for one biological replicate performed in triplicate is shown. Error bars represent the s.d.

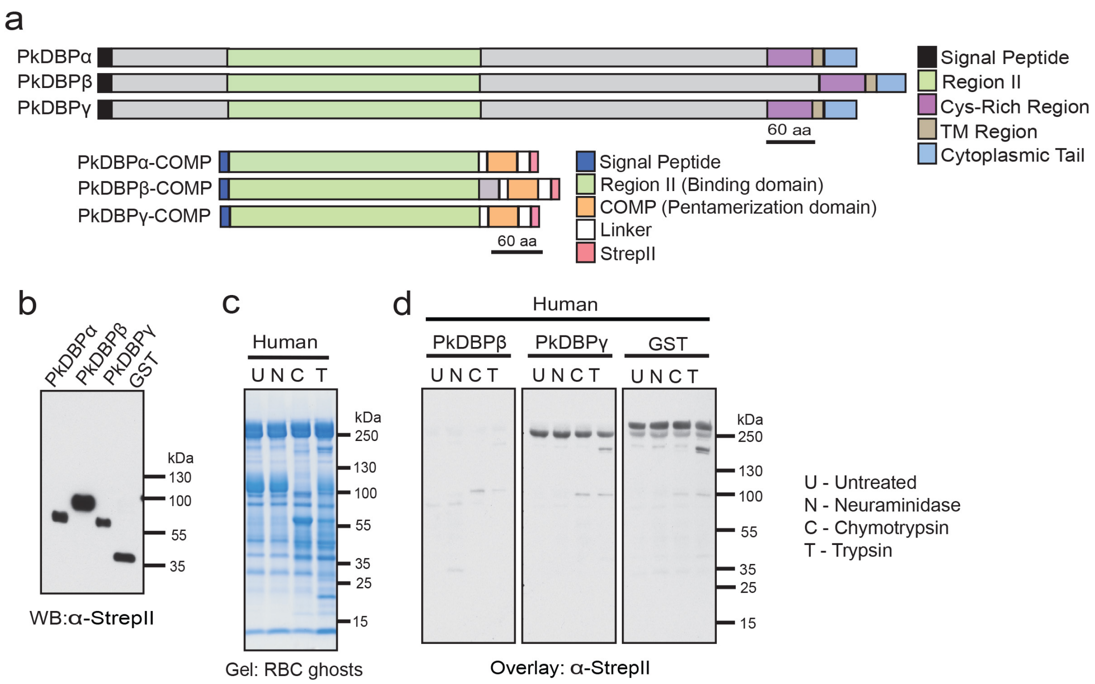

Supplementary Figure 5. Domain structures, expression of PkDBPα, PkDBPβ and PkDBPγ and lack of binding of PkDBPβ and PkDBPγ to human RBCs. (**a**) Domain structures of native PkDBPα, PkDBPβ and PkDBPγ and expression constructs of the Duffy binding domain (Region II) of each protein. (**b**) Western blot showing expression of strepII-tagged PkDBPα-COMP, PkDBPβ-COMP, PkDBPγ-COMP and GST-COMP protein. (**c**) Coomassie-stained protein gel of enzyme-treated and untreated human RBC ghosts. U – untreated, N – neuraminidase-treated, C – chymotrypsin-treated, T - trypsin-treated. (**d**) PkDBPβ-COMP, PkDBPγ-COMP and GST-COMP control protein do not bind to untreated or enzyme-treated human RBC ghosts. Data are representative of at least 4 independent experiments. (See Fig. 3b for binding to rhesus macaque RBC ghosts.)

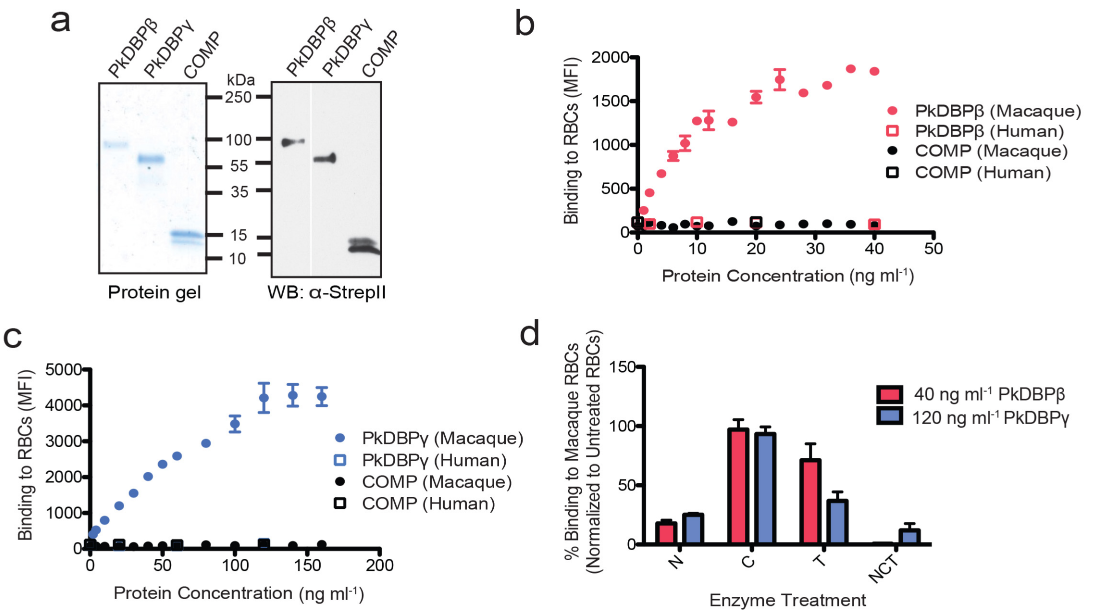

Supplementary Figure 6. Dose-dependent and saturable binding of PkDBPβ and PkDBPγ to rhesus macaque RBCs. (**a**) Coomassie-stained protein gel (Left), and α-StrepII Western blot (Right) of purified PkDBPβ-COMP, PkDBPγ-COMP and COMP protein. (**b**,**c**) Binding of PkDBPβ-COMP (**b**; red circles) and PkDBPγ-COMP (**c**; blue circles) to macaque RBCs in solution increases to the point of saturation with increasing concentration of the respective ligand. COMP protein (black) does not bind to RBCs and neither PkDBPβ nor PkDBPγ binds to human RBCs (red, blue open squares). The flow cytometry-based assay, representative of two biological replicates, was seeded in triplicate; error bars represent the s.d. (**d**) Binding of saturating concentrations of PkDBPβ-COMP and PkDBPγ-COMP to untreated and enzyme-treated macaque RBCs. The mean fluorescence intensity (MFI) was determined for binding of PkDBPβ-COMP and PkDBPγ-COMP to enzyme-treated macaque RBCs, then normalized to 'no protein' controls and further normalized to untreated macaque RBCs. Shown is the average of three biological replicates, performed in triplicate. Error bars represent the s.e.m. N – neuraminidase, C – chymotrypsin, T – trypsin, NCT – neuraminidase, chymotrypsin, trypsin.

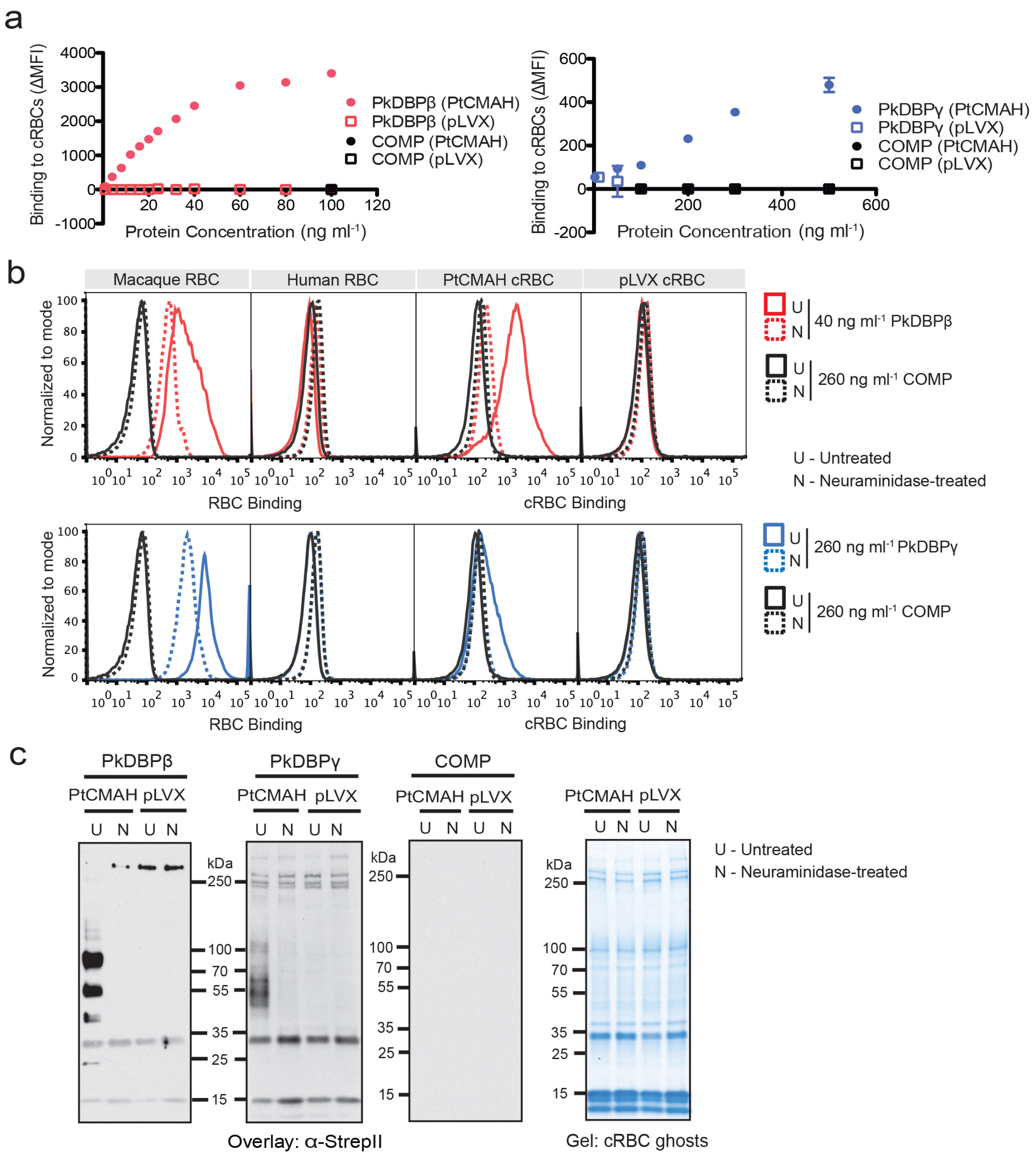

Supplementary Figure 7. PkDBPβ and PkDBPγ bind to Neu5Gc-sialylated receptors. (**a**) Dose-dependent binding of PkDBPβ-COMP (red; left panel) and PkDBPγ-COMP (blue; right panel) to PtCMAH cRBCs (circles) but not pLVX cRBCs (squares). The assay was seeded in duplicate; error bars indicate the range. (**b**) PkDBPβ-COMP (red; top panel) and PkDBPγ-COMP (blue; bottom panel) bind to untreated (U; solid trace) PtCMAH cRBCs and rhesus macaque RBCs, but not to pLVX cRBCs or human RBCs. Binding to neuraminidase-treated (N; dashed trace) PtCMAH cRBCs and macaque RBCs is greatly decreased. COMP protein (black) does not bind to any cell type. Each binding condition was set up in triplicate; a representative trace is shown. The binding assay was done in 2 biological replicates (see Fig. 3c). (**c**) Left – Protein overlays showing binding of PkDBPβ-COMP and PkDBPγ-COMP to PtCMAH, but not pLVX cRBC ghosts or neuraminidase-treated PtCMAH cRBC ghosts. Right – Coomassie-stained protein gel showing protein from cRBC ghost samples used in protein overlays.

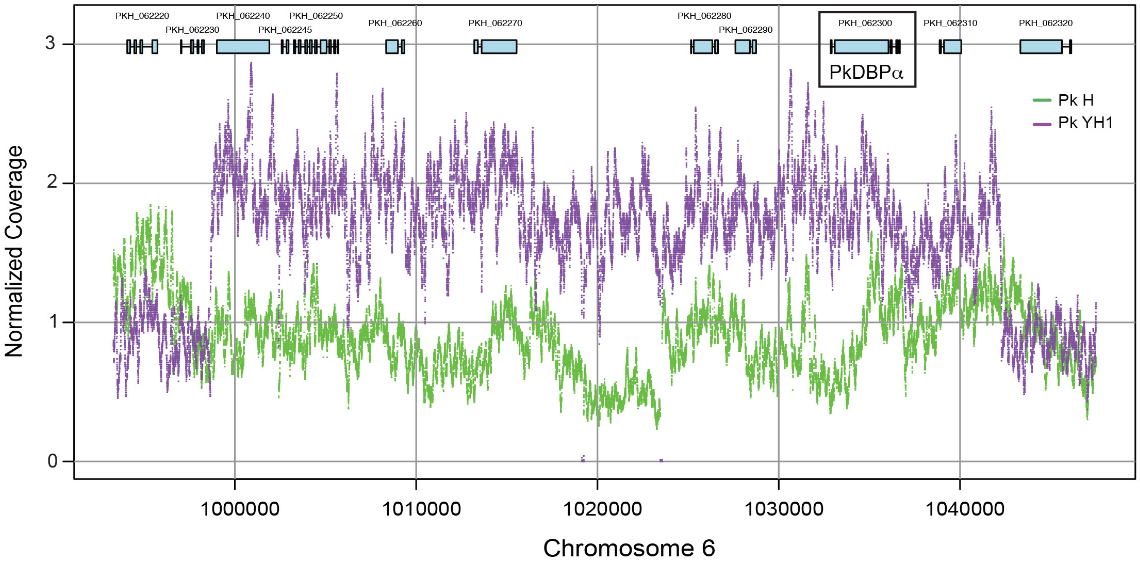

Supplementary Figure 8. Duplication of a region of chromosome 6 containing PkDBPα in the Pk YH1 human-adapted strain. Shown is the sequence read coverage normalized to the average genome-wide read coverage for Pk YH1 and the parental Pk H strain aligned against the *P. knowlesi* H reference genome. The normalized read coverage for the duplicated region is 2. The genes within the depicted region are shown above the corresponding genomic sequence.

Supplementary Table 1. Sialic acid content of red blood cell membranes

		Sialic Acid[b]				
Experiment[a]	RBC Sample	% Neu5Gc	% Neu5Ac	Neu5Gc (pmol)	Neu5Ac (pmol)	Total (pmol)
1	PtCMAH	61.9	38.1	130.6	80.4	211.0
2	PtCMAH	80.8	19.2	225.8	53.7	279.5
3	PtCMAH	74.2	25.8	372.0	129.1	501.0
1	pLVX	ND[c]	100.0	ND	159.8	159.8
2	pLVX	ND	100.0	ND	175.4	175.4
3	pLVX	ND	100.0	ND	319.6	319.6
1	Human	ND	100.0	ND	262.3	262.3
2	Human	ND	100.0	ND	170.7	170.7
3	Human	ND	100.0	ND	271.9	271.9
1	Macaque	98.9	1.1	155.1	1.7	156.8
2	Macaque	100.0	ND	235.7	ND	235.7
3	Macaque	93.2	6.8	325.1	23.7	348.8

[a]Biological Replicate
[b]Sialic acid was released from red blood cell ghosts prepared from $1x10^7$ cells by mild acid hydrolysis, derivatized by reaction with the fluorogenic agent, 1,2-Diamino-4,5-methylenedioxybenzene, and analysed by HPLC. Sialic acid quantity was estimated from Neu5Ac and Neu5Gc standards.
[c]ND – Not detected by HPLC

Supplementary Table 2. Genes in the duplicated region of chromosome 6 in Pk YH1

Gene ID[a]	Gene Name[a]	Signal Peptide?[b]	Transmembrane Prediction[c]
PKH_062230	antigen UB05, putative	No	2 (15-37, 58-80)
PKH_062240	GDP dissociation inhibitor, putative	No	0
PKH_062245	conserved Plasmodium protein, unknown function	No	0
PKH_062250	conserved Plasmodium protein, unknown function	No	0
PKH_062260	Plasmodium exported protein, unknown function, pseudogene	No	1 (35-54)
PKH_062270[d]	erythrocyte binding protein, putative	No	1 (40-59)
PKH_062280	KIR-like protein	No	1 (333-355)
PKH_062290	Plasmodium exported protein, unknown function	No	1 (44-66)
PKH_062300	erythrocyte binding protein (alpha) (PkDBPα)	Yes	1 (1009-1031)
PKH_062310	Plasmodium exported protein, unknown function	Yes	3 (4-21, 276-295, 300-322)

[a]Obtained from PlasmoDB

[b]Signal peptide prediction using locally installed SignalP 4.1.

[c]Transmembrane prediction using locally installed TMHMM 2.0. The amino acids spanning each transmembrane region are in parentheses.

[d]Of the seven predicted membrane proteins, apart from PkDBPα, only PKH_062270 is annotated as an erythrocyte binding protein. This protein, however, has a PEXEL motif, designating it as an exported protein and making it unlikely to play a role in invasion.

Supplementary Table 3. Primer sequences

Gene	Gene ID [a]	Primer	Sequence
CMAH	AF074481.1	Fwd[b]	ggaggGGATCCCAAGCTTGAATTCGCCACCATGG
		Rev[c]	ggaggTCTAGAATCGATTTCGAATCATCACAGGTCCTC
Pk ssuD	PKH_050112	Fwd	gcgcGGATCCCTTGTCTCAAAGATTAAGCCATGC
		Rev	gcgcGTCGACTTCACCTACGGAAACCTTGTTACG
PkDBPα	PkH_062300	Fwd	ggaggGCTAGCGTTATTAATCAAACTTTTCTTCAAAACAATGTAATGGACAAGTGTAATGATAAGAGAAAACG
		Rev	ggaggGCGGCCGCACCTGATTGAGAACCTGGATCAGCTTCATTTGTGCTAGACTTACCTTCACCTTTG
PkDBPβ	PkH_000490	Fwd	ggaggGCTAGCGTTATTAATCAAACTTTTCTTCAGAAGAATGTAATGAGGAGG
		Rev	ggaggGCGGCCGCGAATTTATACGTATCAACATTTTTGGCAGCC
PkDBPγ	PkH_134580	Fwd	ggaggGCTAGCGCTATTAATCAAATTTTTCTTCAAAACAATGTAATGG
		Rev	ggaggGCGGCCGCACCTGGTTGGGAACCTGG
GST		Fwd	ggaggGCTAGCTCCCCTATACTAGGTTATTGGAAAATTAAG
		Rev	ggaggGCGGCCGCATCCGATTTTGGAGGATGGTC

[a]Obtained from GenBank (CMAH) or PlasmoDB (*P. knowlesi* genes)
[b]Forward
[c]Reverse
